# The Network of Exosomes miRNA and p‐MLC2 Regulatory Pathway Induced Pathological Cardiac Hypertrophy in Vasn Deficient Mice

**DOI:** 10.1111/jcmm.70929

**Published:** 2025-11-14

**Authors:** Bin Huang, Qiurui Li, Siwei Yin, Jun Zhang, Xiaoping Guo, Na Yu, Bing Hu, Lanyu Li, Qiaojuan Huang, Min He, Junming Sun

**Affiliations:** ^1^ Affiliated Hospital of Youjiang Medical University for Nationalities Baise Guangxi China; ^2^ Key Laboratory of Research and Development on Clinical Molecular Diagnosis for High‐Incidence Diseases of Baise Baise Guangxi China; ^3^ Laboratory Animal Center Guangxi Medical University Nanning Guangxi China; ^4^ Laboratory Animal Center of Guilin Medical University Guilin Guangxi China; ^5^ Guangxi Key Laboratory of Diabetic Systems Medicine Guilin Medical University Guilin Guangxi China; ^6^ Department of Cardiology, the Second Affiliated Hospital Guangxi Medical University Nanning Guangxi China; ^7^ School of Public Health Guangxi Medical University Nanning China; ^8^ Ministry of Education, Key Laboratory of High‐Incidence‐Tumor Prevention & Treatment Guangxi Medical University Nanning China

**Keywords:** exosomes, mice, miRNA, pathological cardiac hypertrophy, p‐MLC2, signalling pathway, vasorin

## Abstract

Pathological cardiac hypertrophy was an important inducement of heart failure, cardiac arrest, and other diseases. To explore how Vasn knockout induced pathological cardiac hypertrophy, bioinformatics and functional studies illustrated the possible mechanism by clarifying the influence of exosome miRNA on the p‐MLC2 signal pathway. B‐ultrasound, electrocardiogram, pathological staining, and Q‐PCR were used to clarify the changes in typical imaging indexes, pathological indexes, and marker molecules. Exosome sequencing and bioinformatics analysis were carried out to mine key miRNA and signal pathways. Q‐PCR, IHC, and WB were used to verify the changes in miRNA and related signal pathways. The changes in heart structure and function were detected by pathological staining, electron microscopy, B‐ultrasound, and blood biochemistry in the heart tissues and blood of Vasn knockout mice. Vasn knockout mice showed typical imaging, pathological, and molecular features of PCH. Differential analysis of exosome miRNA showed that let‐7g‐5p, let‐7f‐5p, and miR‐148a‐3p significantly increased in the exosomes of Vasn‐knockout mice heart. Bioinformatics analysis showed that let‐7g‐5p and let‐7f‐5p targeted the Calm/MLCK/p‐MLC2 signal pathway, and miR‐148a‐3p targeted the Rhoa/ROCK1/p‐MLC2 signal pathway. The expression levels of miRNA were significantly up‐regulated, but related proteins of signal pathways were significantly reduced in Vasn knockout mice. The structure and function showed obvious damage in Vasn knockout mice. VASN knockout led to pathological cardiac hypertrophy, which may regulate the p‐MLC2 signalling pathway through exosomal miRNA.

## 1 Introduction

Pathological cardiac hypertrophy (PCH) was characterised by an increase in cardiac cell volume and intercellular matrix, accompanied by cardiac cell apoptosis and necrosis, and was an important cause of dangerous events such as arrhythmia, heart failure and sudden death [1, 2]. Vasorin (Vasn), also known as slit‐like 2 (slitl2), contained two exons, of which exon 2 was the main coding region [3]. Vasn was a transmembrane glycoprotein composed of 673 amino acids, which was located on the cell surface [4]. Vasn was highly expressed in the cardiovascular system such as the heart, vascular smooth muscle and umbilical vein endothelial cells [3, 4]. The up‐regulation of Vasn expression could prevent smooth muscle cell calcification by specifically binding to transforming growth factor [5]. The down‐regulation of Vasn expression could alleviate the adverse reaction of vascular wall injury [6]. However, overexpression or knockout of Vasn caused developmental abnormalities in the heart and blood vessels in zebrafish [7]. Vasn knockout mice died suddenly after 3 weeks of birth [8]. Our previous study reported that the Vasn systematic knockout (*Vasn* ^−/−^) model mice showed PCH and cardiac fibrosis symptoms [8, 9].

Exosomes were extracellular vesicles that resembled spherical or cup‐shaped shapes, with a diameter between 30 and 150 nm. Exosomes included proteins, DNA, mRNA and microRNAs that regulated intercellular signalling [10]. Exosomes can be used as potential markers of cardiovascular injury because of the different contents released by cardiac muscle cell [11]. Exosomes played an important role as paracrine and autocrine factors in the repair of cardiac injury [12]. Exosomes were involved in the pathogenesis of cardiac hypertrophy [10], heart failure [13], cardiac cell apoptosis [14], and myocardial fibrosis [15]. MicroRNA (miRNA), as a type of endogenous short‐stranded non‐coding RNA, composed of approximately 20–25 nucleotides, targeted the 3′ UTR terminal nucleotides of mRNA binding and participated in the transcription and post‐transcriptional regulation of target genes [16]. Exosomal miRNAs affected PCH by inhibiting factors related to cardiac cell signalling pathways or reducing mRNA expression of specific genes [17]. Exosomal miRNAs also induced PCH by specifically binding to related mRNA in the heart tissue of normal animals [18]. Exosomal miRNAs could postpone the development of PCH and improve cardiac function through specific binding to targeted mRNA [19].

In this study, Vasn systematic knockout mice showed typical imaging, pathological and molecular features of PCH. The exosomes of mice heart tissue were extracted, identified and sequenced in vitro. Bioinformatics analysis showed that the expression of key exocrine miRNA significantly increased. Exosomes miRNA targeted the Calm/MLCK/p‐MLC2 and the Rhoa/ROCK1/p‐MLC2 signal pathway. The expression levels of miRNA were significantly up‐regulated, but related proteins of these signal pathways were significantly reduced in the heart tissue of Vasn knockout mice. Our results revealed that miRNA‐mRNA networks regulated p‐MLC2 signalling pathway.

## 2 Materials and Methods

### 2.1 Preparation and Blood Biochemistry of Vasn Knockout Mice

C57BL/6J mice were obtained from the Laboratory Animal Center of Guangxi Medical University [SCXK GUI 2020–0003, SYXK GUI 2020–0004]. Vasn^−/−^ mice were produced by inbreeding with *Vasn* ^+/−^ mice. Each mouse genotype was identified by PCR amplification and electrophoresis according to the previous protocol [8, 9]. When *Vasn* ^−/−^ mice approached 28 days old, they exhibited behaviours and morphological characteristics such as an arched back, sparse hair, reduced body size and immobility. *Vasn* ^+/+^, *Vasn* ^+/−^and *Vasn* ^−/−^ mice from the same batch were divided into three groups for subsequent experiments. Blood biochemistry was performed according to the previous protocol [8, 9].

### 2.2 Imaging Analysis

Imaging diagnostic methods included electrocardiographic testing and ultrasound examination used by the BL‐420 N biological function experimental system and the Visual Sonics Vevo 2100 ultrasound. All mice were anaesthetised with 3% pentobarbital sodium. Mice limbs were inserted by probes according to the BL‐420 N operation process. The changes in the heart P wave, PR interval, QRS complex, ST segment, T wave, U wave and QT interval were recorded. Cardiac images were collected along the long axis of the left sternum. The changes in stroke output, ejection fraction (EF), left ventricular anterior and thick wall thickness (LVAWd, LVAWs, LVPWd, LVPWs), left ventricular inner diameter (LVIDd, LVIDs), and other information were recorded and analysed.

### 2.3 Pathological Analysis of HE, Masson and Sirius Red Staining

HE staining was performed according to the previous protocol [8, 9]. Masson staining was performed according to the previous protocol [9]. Sirius Red staining was performed according to the previous protocol [9].

### 2.4 Isolation, Identification and miRNA Sequencing of Exosomes

Mice were subjected to cardiac perfusion using 1 × PBS under anaesthesia. The isolated heart was repeatedly washed with PBS, digested overnight at 4°C in 2.4 U/mL of Dispase II, cut into approximately 1 mm^3^, and placed in a 50 mL centrifuge tube. Tissue fragments were added 5 times the volume of 0.2% type I collagenase solution and digested at 37°C for 1.5 h. The tissue fluid was gently blown to form a single‐cell suspension. The suspension was added to PBS to the appropriate volume and centrifuged at 2000 *g* at 4°C for 10 min. The supernatant was centrifuged at 10,000 *g* at 4°C for 45 min and filtered by 0.45 μM membrane filtration. The filtrate was centrifuged at 100,000 *g* at 4°C for 70 min, and the supernatant was removed. Exosomes were resuspended with 10 mL of precooled PBS. Extracted exosomes were stored at −80°C. The exosomes were sequenced according to the small RNA sequencing process. The raw data of exosomes miRNAs excluded some reads with 5′ connectors, a length of less than 18 bp, and low quality. The computer conducted distribution statistics on the length of small RNAs. The software conducted differential expression analysis of miRNAs between samples (at least 2 or more samples) and then predicted the target genes of miRNAs. The systems software performed GO/KEGG functional enrichment and analysis based on miRNA target genes.

### 2.5 The Primary Isolation and Culture of Cardiomyocytes

Three‐day‐old mice were anaesthetised by intraperitoneal injection of 3% pentobarbital sodium. These hearts were perfused at a speed of about 4 mL/min, perfused with D‐Hank's buffer, perfused with 1.5 mg/mL collagenase II buffer, and then perfused with a buffer containing 1 mol/L CaCl_2_. When the heart became bigger, white and transparent, the heart tissue was fully cut. The heart tissue stopped the digestion by adding buffer. The cell suspension was gently blown repeatedly, filtered with filter paper, and centrifuged twice at a speed of 1000 r/min, each time for 1 min. The precipitate was added with buffer and placed in a 60 mm Petri dish, and the number of rod‐shaped cardiomyocytes was adjusted to 25,000/mL, and the complete culture medium of mouse cardiomyocytes was added.

### 2.6 Quantitative PCR Analysis

RNA reverse transcription and quantitative PCR was performed according to the previous protocol [8, 9]. These primers (Table 1) were produced by Sangon Biotech (Shanghai). Each miRNA and gene was subjected to 40 cycles of PCR, and this process was repeated at least three times. The expression levels of the endogenous miRNA *U6* and gene *GAPDH* were used for comparison. The relative expression of target miRNAs and genes was compared by using the 2^–△△CT^ method.

**FIGURE 1:**
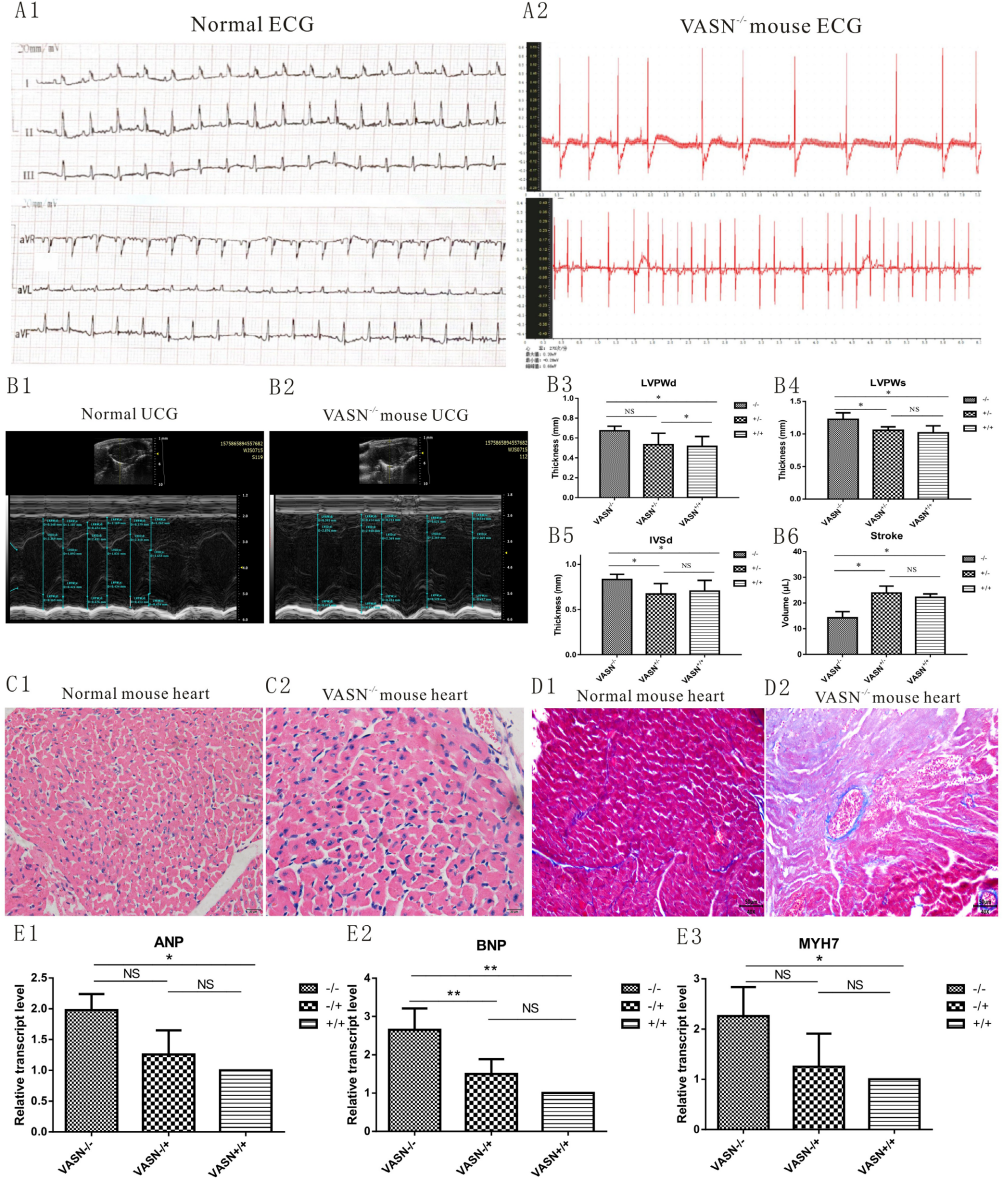
Changes in diagnostic indicators of PCH in Vasn knockout mice. A1 and A2: Changes of the electrocardiogram in Vasn mice; A1 was a normal electrocardiogram including I, II, III, aVR, aVL and aVF leads; A2 was abnormal electrocardiogram. B1 and B2: Changes of the echocardiogram in Vasn mice; B1 was the normal echocardiogram of Vasn^+/+^ and Vasn^+/−^ mice; B2 was the abnormal echocardiogram of Vasn^−/−^ mice. B3 to B6: Changes of the ultrasound indicators in Vasn mice. C1 and C2: Changes of the HE staining in Vasn mice; C1 was the normal structure of Vasn^+/+^ and Vasn^+/−^ mice; C2 was the abnormal structure of Vasn^−/−^ mice. D1 and D2: Changes of the Masson staining in Vasn mice; D1 was the normal structure of Vasn^+/+^ and Vasn^+/−^ mice; D2 was the abnormal structure of Vasn^−/−^ mice. E1 to E3: Changes of the molecular markers in Vasn mice. *p* < 0.05 indicated significant differences, *p* < 0.01 indicated extremely significant differences, and the sub tables were represented by superscripts * and * *.

**TABLE 1:** List of primer sequences.

Genes	Forward/Reverse	Sequences
*let‐7f‐5p*	Forward Reverse	CGCGCGTGAGGTAGTAGATTGT AGTGCAGGGTCCGAGGTATT
*miR‐148a‐3p*	Forward Reverse	GCGCGTCAGTGCACTACAGAA AGTGCAGGGTCCGAGGTATT
*let‐7g‐5p*	Forward Reverse	CGCGCGTGAGGTAGTAGTTTGT AGTGCAGGGTCCGAGGTATT
*miR‐145a‐3p*	Forward Reverse	CGCGATTCCTGGAAATACTG AGTGCAGGGTCCGAGGTATT
*U6*	Forward Reverse	CTCGCTTCGGCAGCACA AACGCTTCACGAATTTGCGT
*Rock1*	Forward Reverse	TGAAAGCCGCACTGATGGAT TCATTCCAGCCATGAGAAAATACT
*Calm4*	Forward Reverse	CCTTGAGCACTGGACCAGAAA TTGAAAGCTGCCTGGAACTC
*Calm3*	Forward Reverse	TGGCCAGGTCAATTATGAAGAGTT GTACGCAGGGGAGTGTTGAA
*Rhoa*	Forward Reverse	CGTCGGTTCTCTCCATAGCC TCAGATGCAAGGCTCAAGGC
*MYPT1*	Forward Reverse	CAGTGACCATTCCTGTGGCT GGAGTGAGGTATGACCTGCG
*MYLK3*	Forward Reverse	GCGCTGCTAGATTTGACTGC TCTATCTGGAAGTGGCGGGA
*MYL7*	Forward Reverse	AGGAAGCCTTCAGCTGCATT ACGGTGAAGTTGATGGGACC
*MYL2*	Forward Reverse	CTTCACCGTGTTCCTCACGA TTGTGTGGTCAGCATCTCCC
*GAPDH*	Forward Reverse	CCTCGTCCCGTAGACAAAATG TGAGGTCAATGAAGGGGTCGT

### 2.7 WB Analysis

Western blot was performed according to the previous protocol [8, 9]. Primary antibodies including ROCK1 (D221198, Sangon Biotech, 1:300), CALM3 (A14526, ABclonal, 1:300), RHOA (A0272, ABclonal, 1:300), MYPT1 (D222722, Sangon Biotech, 1:300) and endogenous protein β‐ACTIN (66009–1‐Ig, Proteintech, 1:500) were used. The secondary antibody was horseradish peroxidase (HRP)–conjugated (AS014, ABclonal, 1:1000). The expression level of the target protein was calculated by an automatic analysis system (Image Lab 6.0).

### 2.8 Immunohistochemical and Immunofluorescence Analysis

IHC‐P and IF were performed according to the previous protocol [8, 9]. Primary antibody against ROCK1 (D221198, Sangon Biotech, 1:500), CALM3 (A14526, ABclonal, 1:500), RHOA (A0272, ABclonal, 1:500), MYPT1 (D222722, Sangon Biotech, 1:500), p‐MLC2 (3671 T, CST, 1:300) and HRP secondary antibody (AS014, ABclonal, 1:500) were used.

### 2.9 Electron Microscope Analysis

Electron microscopy was performed according to the previous protocol [8, 9]. Exosomes were identified through electron microscopy. About 10 μL of exosomes were dropped onto a copper mesh and precipitated for 1 min, and the floating liquid was removed by suction with filter paper. About 10 μL of uranium acetate was dropped onto a copper mesh and precipitated for 1 min, and the floating liquid was removed by suction with filter paper. The exosomes were dried at room temperature for a few minutes and then observed under electron microscopy.

### 2.10 Statistical Analysis

All experiments were repeated at least three times. The data from B‐ultrasound were repeated at least 6 times. All data were presented as the mean and standard deviation. The data were statistically analysed using one‐way ANOVA in SPSS. *p* < 0.05 indicated significant differences, and *p* < 0.01 indicated extremely significant differences.

## 3 Results

### 3.1 Vasn Knockout Mice Showed Pathological Cardiac Hypertrophy

The electrocardiogram showed no obvious abnormalities in the waveforms of *Vasn* ^+/+^ and *Vasn* ^+/−^ mice (Figure 1A1), but the Vasn^−/−^ mice showed common PCH waveforms such as atrioventricular block, left atrial hypertrophy, ST segment depression, T‐wave inversion, and ST‐T changes (Figure 1A2). The ultrasound images showed no abnormalities in various indicators of *Vasn* ^+/+^ and *Vasn* ^+/−^ mice (Figure 1B1), while the distance between peaks and valleys was significantly increased in *Vasn* ^−/−^ mice (Figure 1B2). The LVPWd, LVPWs and IVSd of Vasn^−/−^ mice were significantly thickened, but the stroke volume of *Vasn* ^−/−^ mice was significantly reduced (Figure 1B3–B6). HE staining showed no abnormalities in the heart tissues of *Vasn* ^+/+^ and *Vasn* ^+/−^ mice (Figure 1C1), but a significantly increased area was observed in cardiac cells (Figure 1C2). Masson staining showed no abnormalities in the heart tissues of Vasn^+/+^ and *Vasn* ^+/−^ mice (Figure 1D1), but cardiac interstitial fibrosis was significantly enhanced in Vasn^−/−^ mice (Figure 1D2). The expression of *ANP*, *BNP* and *MYH7* was significantly increased in *Vasn* ^−/−^ mice (Figure 1E1–E3). These results confirmed that Vasn knockout mice exhibited typical PCH symptoms.

### 3.2 Bioinformatics Analysis of Cardiac Exosomes miRNA in Vasn Knockout Mice

**FIGURE 2:**
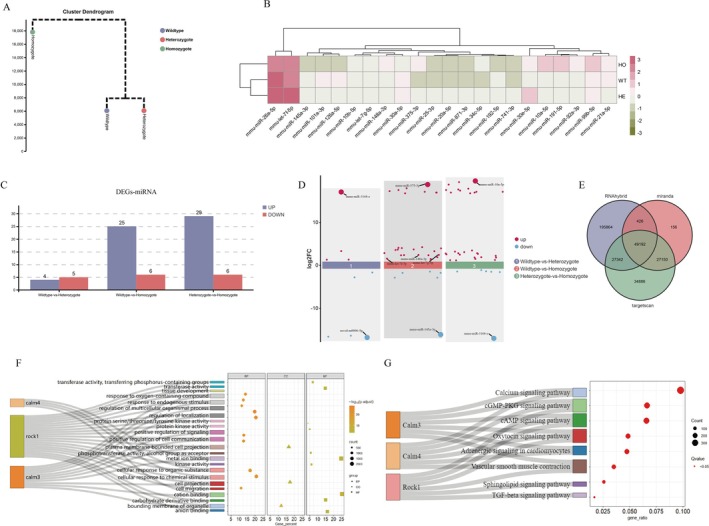
Transcriptome analysis of cardiac exosomes miRNA in Vasn knockout mice. This study selected cardiac exosomes from Vasn^+/+^ mice (*n* = 12), Vasn^+/−^ mice (*n* = 10), and Vasn^−/−^ mice (*n* = 8) for miRNA sequencing. A: Hierarchical clustering chromatography based on miRNA transcriptome data; B: The heat map of target miRNA expression obtained by pairwise comparison; C: Histogram of differential miRNA expression levels amongst different comparison groups; D: Scatter plot of differences amongst comparison groups; E: Wayne plot in the number of miRNA target genes; F: GO enrichment analysis of target miRNA genes; G: KEGG enrichment analysis of target miRNA genes.

In hierarchical clustering chromatography, it was found that the *Vasn* ^+/−^ and *Vasn* ^−/−^ groups were clustered, while the *Vasn* ^+/+^ group was independent, indicating that the three groups were independently grouped (Figure 2A). The differential miRNAs were summarised in three groups, and 22 miRNAs were screened as target miRNAs. Their target genes were obtained by systems software (Figure 2B). Compared to the *Vasn* ^+/+^ group, the *Vasn* ^−/−^ group had 25 upregulated genes and 6 downregulated genes. Compared to the *Vasn* ^+/−^ group, the *Vasn* ^−/−^ group had 29 upregulated genes and 6 downregulated genes (Figure 2C). Compared to the *Vasn* ^+/+^ group, the expression levels of mmu let‐7g‐5p, mmu let‐7f‐5p and mmu miR‐148a‐3p were higher in the *Vasn* ^−/−^ group, while the expression level of mmu miR‐145a‐3p was the lowest (Figure 2D). 49,192 target genes were obtained by three databases: miranda, RNA hybrid and target sacan (Figure 2E).

Through GO enrichment analysis, Rock1 participated in more enrichment items, followed by Calm3 and Calm4 was the least (Figure 2F and Table 2). Through KEGG enrichment analysis, the CALM/MLCK/p‐MLC2 and RHOA/ROCK/p‐MLC2 signalling pathways were a higher number of enriched genes, which were important signalling pathways involved in the process of cardiac disease (Figure 2G and Table 3). These analyses indicated that *let‐7g‐5p*, *let‐7f‐5p* and *miR‐148a‐3p* targeted *Calm3*, *Calm4* and *ROCK1*, respectively.

**TABLE 2:** Go enrichment.

Description	Group	Gene number	*p*	*p*.adjust
Cation binding	MF	2261	< 0.01	< 0.01
Protein serine/threonine/tyrosine kinase activity	MF	359	< 0.01	< 0.01
Protein kinase activity	MF	448	< 0.01	< 0.01
Phosphotransferase activity, alcohol group as acceptor	MF	513	< 0.01	< 0.01
Metal ion binding	MF	2204	< 0.01	< 0.01
Kinase activity	MF	551	< 0.01	< 0.01
Transferase activity, transferring phosphorus‐containing groups	MF	619	< 0.01	< 0.01
Transferase activity	MF	1321	< 0.01	< 0.01
Carbohydrate derivative binding	MF	1329	< 0.01	< 0.01
Anion binding	MF	1436	< 0.01	< 0.01
Bounding membrane of organelle	CC	1047	< 0.01	< 0.01
Cell projection	CC	1532	< 0.01	< 0.01
Plasma membrane bounded cell projection	CC	1476	< 0.01	< 0.01
Regulation of multicellular organismal process	BP	1771	< 0.01	< 0.01
Regulation of localisation	BP	1832	< 0.01	< 0.01
Tissue development	BP	1236	< 0.01	< 0.01
Positive regulation of signalling	BP	1144	< 0.01	< 0.01
Cellular response to chemical stimulus	BP	1850	< 0.01	< 0.01
Response to endogenous stimulus	BP	1048	< 0.01	< 0.01
Positive regulation of cell communication	BP	1137	< 0.01	< 0.01
Cellular response to organic substance	BP	1502	< 0.01	< 0.01
Cell migration	BP	1010	< 0.01	< 0.01
Response to oxygen‐containing compound	BP	1121	< 0.01	< 0.01

**TABLE 3:** KEGG enrichment.

Pathway	Gene number	*p*	*Q*
Calcium signalling pathway	395	< 0.01	< 0.01
cGMP‐PKG signalling pathway	268	< 0.01	< 0.01
cAMP signalling pathway	266	< 0.01	< 0.01
Oxytocin signalling pathway	196	< 0.01	< 0.01
Adrenergic signalling in cardiomyocytes	191	< 0.01	< 0.01
Vascular smooth muscle contraction	142	< 0.01	< 0.01
TGF‐beta signalling pathway	68	< 0.01	< 0.01
Sphingolipid signalling pathway	104	< 0.01	< 0.01
Wnt signalling pathway	129	< 0.01	< 0.01
PPAR signalling pathway	43	< 0.05	< 0.05
NF‐kappa B signalling pathway	68	< 0.05	< 0.05
Cardiac muscle contraction	65	< 0.01	< 0.05

### 3.3 Cardiac Exosomes miRNA Regulated the Calm and ROCK Signal Pathways

**FIGURE 3:**
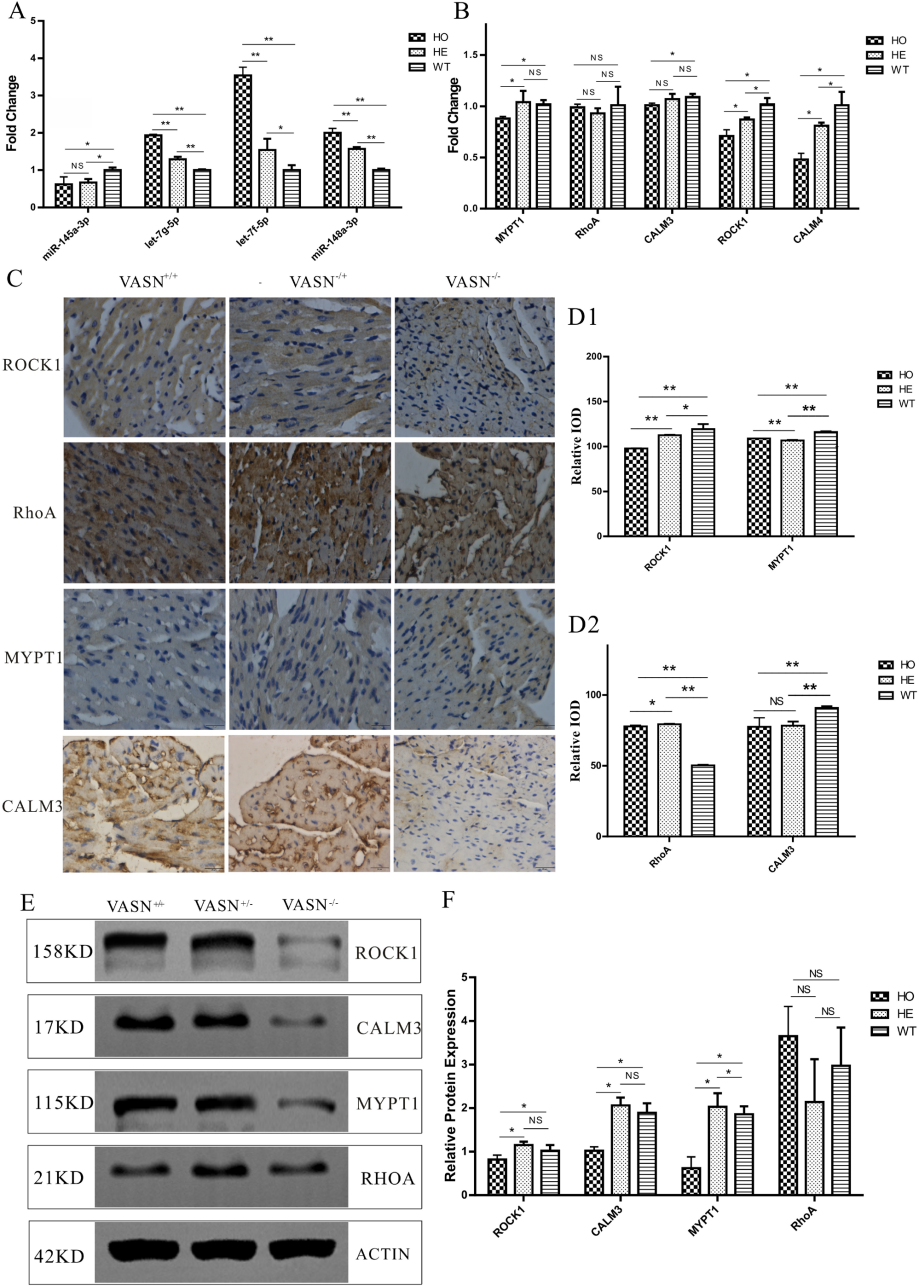
Changes in the key molecules of the signalling pathway in Vasn knockout mice. A: Q‐PCR validation of the expression levels of target miRNAs in cardiac exosomes; B: Q‐PCR validation of the expression levels of target genes in cardiac tissue; C: Immunohistochemical validation of the expression levels of target genes in various signalling pathways; D: WB validation of the expression levels of target genes in various signalling pathways. *p* < 0.05 indicated significant differences, *p* < 0.01 indicated extremely significant differences, and the sub tables were represented by superscripts * and * *.

To investigate whether exosomal miRNA regulated these two signalling pathways, we performed functional validation. The expression levels of *let‐7g‐5p*, *let‐7f‐5p* and *miR‐148a‐3p* were significantly higher in the *Vasn* ^−/−^ exosomes, while the expression level of *miR‐145a‐3p* was significantly lower in the *Vasn* ^−/−^ exosomes (Figure 3A). The mRNA expression levels of *Calm3*, *Calm4*, *Rock1* and *Mypt1* genes were significantly lower in the *Vasn* ^−/−^ heart tissues (Figure 3B). The protein expression levels of Calm3, Rock1 and Mypt1 were significantly lower in the *Vasn* ^−/−^ heart tissues (Figure 3C–F). Combining the results of bioinformatics and functional analysis, the relationship between miRNA and the p‐MLC2 regulatory axis was preliminarily revealed.

### 3.4 The Calm and ROCK Signal Pathways Down‐Regulatedp‐MLC2

**FIGURE 4:**
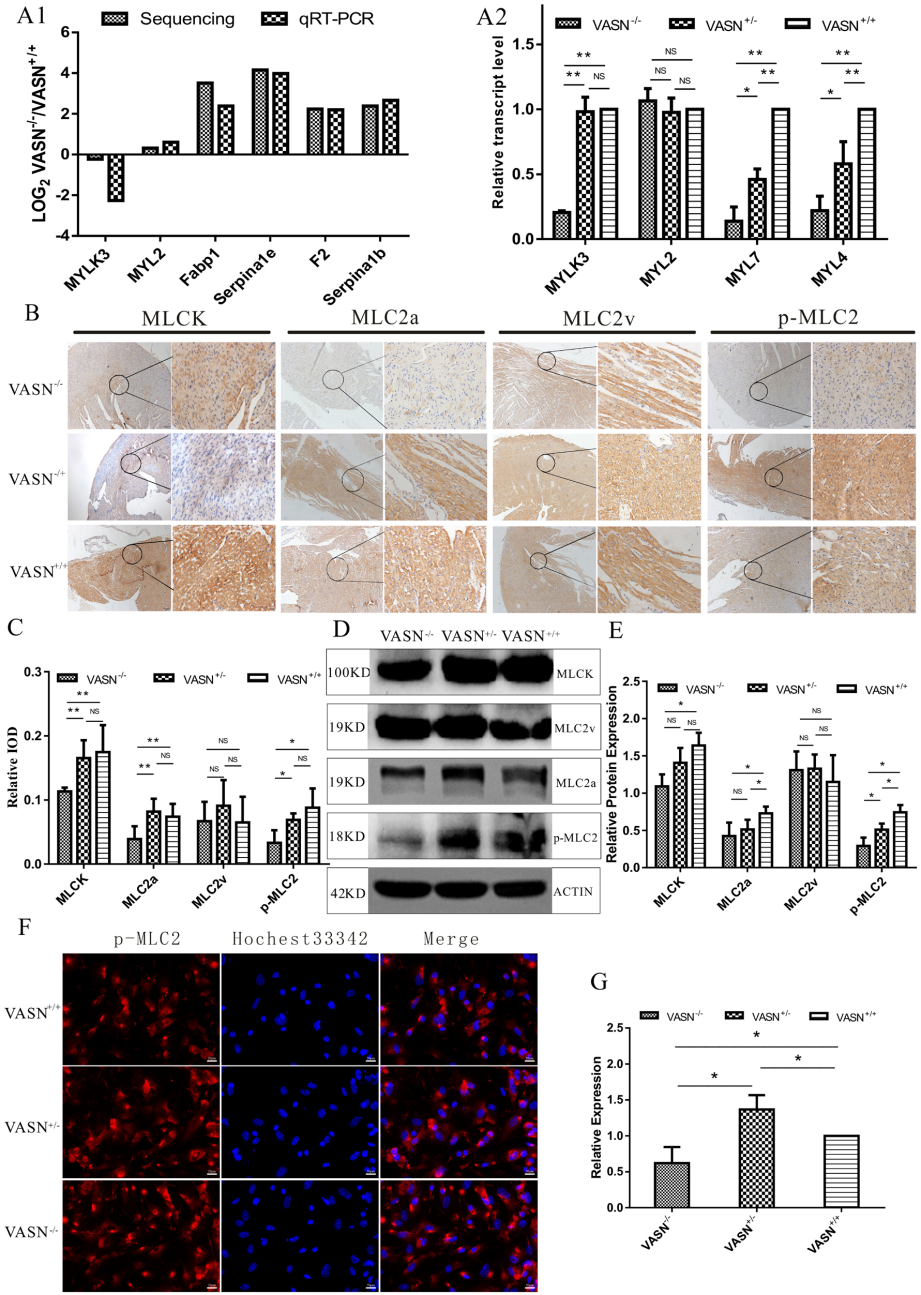
Changes in the key molecules of PCH in Vasn knockout mice. A: Q‐PCR validation of the expression levels of key molecules in cardiac tissue; B: Immunohistochemical validation of the expression levels of key molecules in cardiac tissue; C: WB validation of the expression levels of key molecules in cardiac tissue. *p* < 0.05 indicated significant differences, *p* < 0.01 indicated extremely significant differences, and the sub tables were represented by superscripts * and * *.

To explore whether two signalling pathways regulated the key molecules in PCH, we also performed functional validation. The mRNA expression levels of *MYLK3* and *MYL7* were significantly lower in the *Vasn* ^−/−^ heart (Figure 4A). The protein expression levels of MLCK, MLC‐2a and p‐MLC2 were significantly lower in the *Vasn* ^−/−^ heart tissues (Figure 4B–G). These results indicated that the two signalling pathways directly regulated key molecules in PCH.

### 3.5 Down‐Regulated p‐MLC2 Changed the Structure of Cardiac Fibres

**FIGURE 5:**
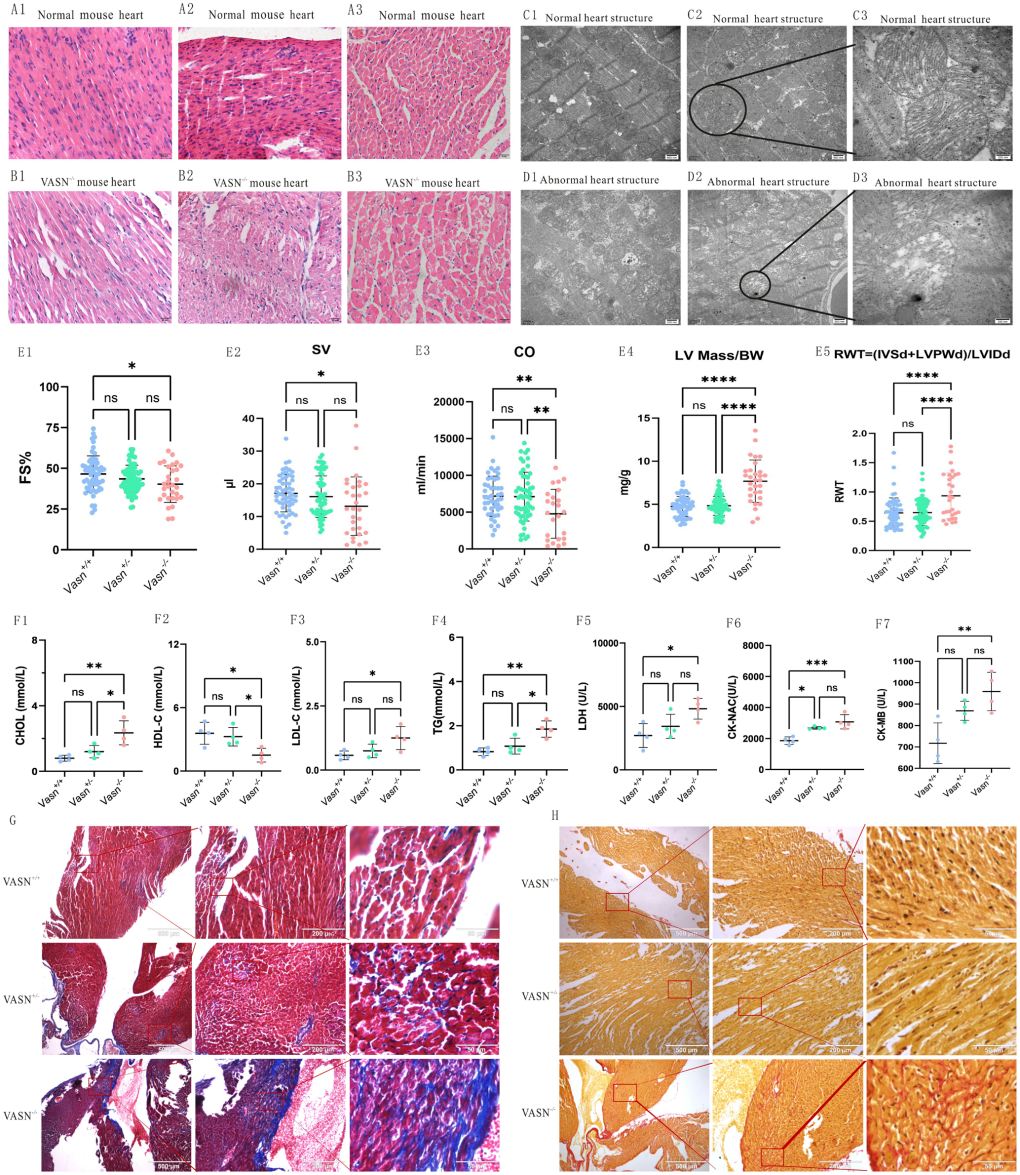
Structural changes of cardiac fibres in pathological cardiac hypertrophy. A1 to A3, B1 to B3: HE staining of cardiac fibres in Vasn mice; A1 and A2 showed regular and tightly spaced of cardiac cells in the longitudinal section, and A3 showed normal and moderately sized cardiac cells in the cross section; B1 and B2 showed cardiac cell rupture, increased gap and a large amount of steatosis in longitudinal section, B3 showed an increase in cardiac cell area, vacuoles and a large amount of steatosis in cross‐section. C1 to C3, D1 to D3: Electron microscope of cardiac fibres in Vasn mice; C1 showed regular and tightly spaced of cardiac fibres, C2 and C3 showed regular mitochondrial morphology; D1 showed abnormal, fractured and irregularly distributed of cardiac fibres, D2 and D3 showed vacuolated and irregularly mitochondrial morphology. E and F: Heart function in Vasn mice; G and H: Heart fibrosis in Vasn mice. *p* < 0.05 indicated significant differences, *p* < 0.01 indicated extremely significant differences, *p* < 0.0001 indicated extremely significant differences, and the sub tables were represented by superscripts *, ** and****. The scale for electron microscope was 500 nm and 200 nm.

**FIGURE 6:**
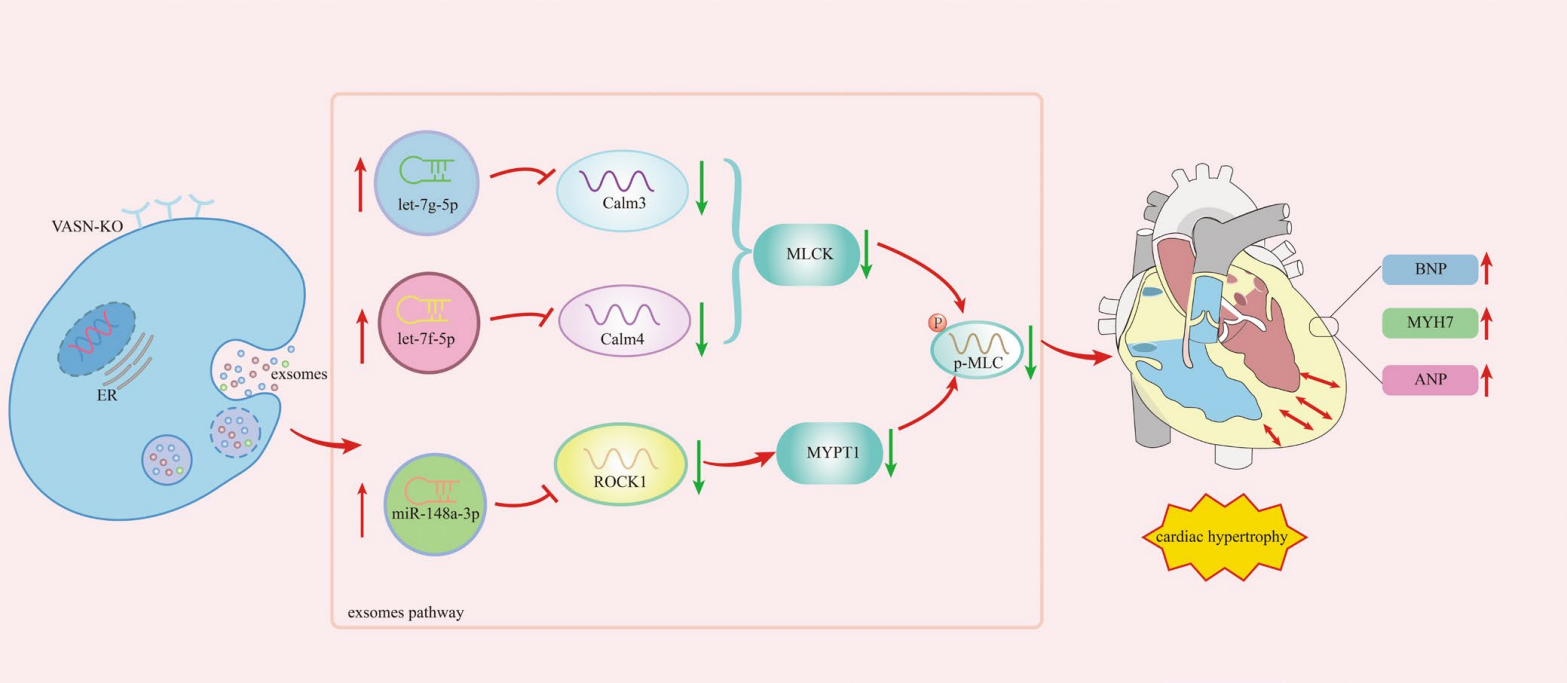
Mechanism diagram which Vasn knockout induces cardiac hypertrophy by regulating the p‐MLC signalling pathway through exosome miRNA. This figure illustrated the molecular mechanism underlying the therapeutic effect of exosome miRNA on cardiac hypertrophy. The diagram began with Vasn knockout, which was localised on the cell membrane. This interaction activated exosome miRNA, as indicated by the arrows pointing from exosome to cardiac cell. miR‐let‐7g‐5p and miR‐let‐7f‐5p targeted the Calm/MLCK signal pathway, and miR‐148a‐3p targeted the Rhoa/ROCK1 signal pathway. The activation of target MLCK and MYPT1 resulted in the reduced expression of p‐MLC2, which was known to counteract the progression of cardiac hypertrophy. The mechanism was summarised in a clear and concise manner, highlighting the key steps involved in the target action of exosome miRNA in Vasn knockout mice.

We detected whether down‐regulated p‐MLC2 induced the abnormalities of myocardial structure. The cardiac fibres of *Vasn* ^+/+^ and *Vasn* ^+/−^ mice showed regular, tightly spaced, and moderately sized in the longitudinal section (Figure 5A1–A3,C1), while the *Vasn* ^−/−^ heart showed severe steatosis, fractured, irregular arrangement, and significant area enlargement (Figure 5B1–B3,D1). The ultra‐microstructure of *Vasn* ^+/+^ and *Vasn* ^+/−^ heart showed regular mitochondrial morphology (Figure 5C2,C3), but the Vasn^−/−^ heart showed vacuolated and irregular mitochondrial morphology (Figure 5D2,D3). The *Vasn* ^−/−^ mice heart function showed seriously damaged (Figure 5E,F). The *Vasn* ^−/−^ mice heart structure showed obvious fibrosis (Figure 5G,H). These may be the structural basis for inducing pathological cardiac hypertrophy (Figure 6, Mechanism diagram).

## 4 Discussion

The clinical manifestation of PCH was the enlargement of cardiac cells and the increase of intercellular matrix, accompanied by cardiac cell apoptosis and necrosis. PCH was also an important trigger for dangerous events such as arrhythmia, heart failure and sudden death. However, the pathogenesis of PCH was not yet fully understood [1]. Diagnostic indicators for PCH included imaging detection, tissue pathology and molecular markers. The combined evaluation of electrocardiogram and echocardiography was an effective imaging diagnostic protocol for PCH [20]. T‐wave inversion, ST wave depression, and bundle branch block were common PCH electrocardiograms, in which ST‐T wave changes were the most common [21]. Ventricular hypertrophy, thickened cardiac chambers, and abnormal blood flow were common PCH echocardiography [22]. Cardiac cell hypertrophy, steatosis, interstitial fibrosis and decreased capillary density were widely observed in HE, Masson and Sirius Red staining. The increased expression levels of typical molecular markers such as atrial natriuretic peptide (ANP), brain natriuretic peptide (BNP) and myosin heavy chain 7 (MYH7) were diagnostic criteria for PCH [23, 24]. Our experimental results were consistent with the above literature; thus, these indicated that Vasn knockout mice exhibited typical PCH symptoms.

Vasn was a transmembrane protein located on the surface of cell membranes and expressed abundantly in cardiovascular systems such as the heart, vascular smooth muscle and umbilical vein endothelial cells. Overexpression or knockout of Vasn caused developmental abnormalities in the heart and blood vessels in zebrafish [25]. Vasn knockout mice suddenly died after 3 weeks of birth [5, 8]. Our previous research firstly found that Vasn knockout induced PCH by downregulating the expression level of MYL7 in mice [8]. MYL7 (MLC2a or RLCa) was a member of the RLC family and was expressed in both the ventricle and atrium [26, 27]. MLC2 (Myosin regulatory light chain 2) was the main component of cardiac myosin regulatory light chains, divided into atrioventricular type (MLC2a) and ventricular type (MLC2v) [28]. Phosphorylation MLC2 (p‐MLC2) affected myocardial structure and function [29, 30]. The p‐MLC2 was a post‐translational modification of proteins involved in the regulation of MLC2 spatial conformation [31]. Cardiac p‐MLC2 was mainly concentrated on Ser15 or Ser14 of MLC2v peptides [32]. The p‐MLC2 enhanced cardiac function, while p‐MLC2 deletion weakened cardiac function [32]. Increasing p‐MLC2 could alleviate adverse emergency reactions caused by PCH [30]. By surgical constriction of the abdominal aorta [33] or the addition of isoproterenol [34], decreasing p‐MLC2 induced PCH symptoms such as cardiac cell hypertrophy, disordered arrangement of sarcomeres, and upregulation of marker genes [31]. Therefore, the downregulation of p‐MLC2 expression leading to cardiac structural abnormalities was an important trigger for PCH.

Exosomes could regulate physiological processes such as cardiac function, myocardial development, angiogenesis and immune regulation [35] through the proteins, mRNA and microRNA, as well as participated in pathological processes such as myocardial hypertrophy [10], heart failure [13], cardiomyocyte apoptosis [14], and myocardial fibrosis [15]. Exosomes' miRNAs played a crucial role in transcriptional regulation by directly targeting important genes or signalling pathways during cardiovascular development, normal physiological function, and related diseases [16, 17, 18]. In our results, differential analysis of exosomes' miRNA showed that miR‐let‐7g‐5p, miR‐let‐7f‐5p and miR‐148a‐3p significantly increased. Exosomes' miR‐let‐7g‐5p and miR‐let‐7f‐5p targeted the Calm/MLCK/p‐MLC2 signalling pathway, and exosomes' miR‐148a‐3p targeted the Rhoa/ROCK1/p‐MLC2 signalling pathway. By quantitative PCR, IHC‐P and WB, the related proteins of these signalling pathways were significantly decreased in the heart tissue of Vasn knockout mice. These results demonstrated for the first time that Vasn knockout caused an increased expression of exosomes' miRNA to regulate the signalling pathway of PCH.

Exosomes miR‐let‐7g‐5p participated in the enhancement of inflammatory response in chronic heart failure [36]. Cardiac miR‐let‐7g‐5p targeted the key signalling pathway in cardiomyocyte development and consequently heart pathophysiology progression [37]. In clinical research on acute myocardial infarction, exosomes miR‐let‐7g‐5p expression was inversely associated with serum levels of pro‐inflammatory cytokines [38]. The expression level of miR‐let‐7f‐5p in serum was closely related to the spontaneous coronary artery dissection [39]. miR‐148a‐3p in myocardial tissue promoted myocardial hypertrophy by targeting key signalling pathways [40]. The multi‐omics analysis revealed miR‐148a‐3p may be considered a potential biomarker for early diagnosis and progression of cardiometabolic diseases [41]. There were no studies that exosomes let‐7g‐5p, let‐7f‐5p and miR‐148a‐3p targeted the Calm/MLCK/p‐MLC2 and Rhoa/ROCK1/p‐MLC2 signal pathway to induce myocardial hypertrophy.

## 5 Conclusions

In summary, Vasn knockout mice showed typical pathological, imaging and molecular features of PCH. The expression of exosomes let‐7g‐5p, let‐7f‐5p and miR‐148a‐3p significantly increased. Bioinformatics analysis predicted exosomes miR‐let‐7g‐5p and miR‐let‐7f‐5p targeted the Calm/MLCK/p‐MLC2 signal pathway, and exosomes miR‐148a‐3p targeted the Rhoa/ROCK1/p‐MLC2 signal pathway. The related proteins of these signal pathways were significantly reduced in the heart tissue of Vasn knockout mice. VASN knockout led to pathological cardiac hypertrophy, which may regulate the p‐MLC2 signal pathway through exosomes miRNA (Figure 6, Mechanism diagram).

## Author Contributions

**Bin Huang:** methodology (equal), project administration (equal), software (equal), visualization (equal). **Qiurui Li:** data curation (equal), formal analysis (equal), project administration (equal), resources (equal), software (equal), validation (equal), writing – original draft (equal). **Siwei Yin:** conceptualization (equal), data curation (equal), formal analysis (equal), methodology (equal), resources (equal), software (equal), visualization (equal), writing – original draft (equal), writing – review and editing (equal). **Jun Zhang:** conceptualization (equal), data curation (equal), formal analysis (equal), resources (equal), software (equal), writing – original draft (equal), writing – review and editing (equal). **Xiaoping Guo:** conceptualization (equal), formal analysis (equal), project administration (equal), resources (equal), validation (equal), writing – original draft (equal), writing – review and editing (equal). **Na Yu:** data curation (equal), resources (equal), software (equal). **Bing Hu:** conceptualization (equal), project administration (equal), resources (equal), software (equal). **Lanyu Li:** conceptualization (equal), formal analysis (equal), investigation (equal), project administration (equal), software (equal), validation (equal), visualization (equal). **Qiaojuan Huang:** conceptualization (equal), funding acquisition (equal), project administration (equal), resources (equal), validation (equal), writing – review and editing (equal). **Min He:** funding acquisition (equal), project administration (equal), resources (equal), validation (equal). **Junming Sun:** conceptualization (lead), data curation (lead), formal analysis (lead), funding acquisition (lead), investigation (lead), methodology (lead), project administration (lead), resources (lead), software (lead), supervision (lead), validation (lead), visualization (lead), writing – original draft (lead), writing – review and editing (lead).

## Ethics Statement

All mice experiments were approved by the ethics committee of Guangxi Medical University [201909116].

## Consent

The authors have nothing to report.

## Conflicts of Interest

The authors declare no conflicts of interest.

## Data Availability

All data and materials were valid.
